# USE OF SERVICES BY PEOPLE LIVING ALONE WITH COGNITIVE IMPAIRMENT: A SYSTEMATIC REVIEW

**DOI:** 10.1093/geroni/igz038.3312

**Published:** 2019-11-08

**Authors:** Amy Rosenwohl-Mack, Anna Chodos, Sarah Dulaney, Min-Lin Fang, Jennifer Merrilees, Leslie Dubbin, Elena Portacolone

**Affiliations:** 1 University of California, San Francisco, San Francisco, California, United States; 2 Memory and Aging Center, University of California, San Francisco, San Francisco, California, United States; 3 Education and Research Services, UCSF Library, University of California, San Francisco, San Francisco, California, United States; 4 Department of Social & Behavioral Sciences, School of Nursing, University of California, San Francisco, San Francisco, California, United States

## Abstract

At least one third of older adults with dementia live alone in the United States. Living alone may represent an opportunity to maintain independence and autonomy, while remaining in a familiar home environment. However, living alone with cognitive impairment is also associated with health risks and unmet needs. No systematic reviews on this population have been published. We systematically reviewed research on use of healthcare and long-term services and supports (LTSS) by people living alone with cognitive impairment. Following PRISMA guidelines, we searched six electronic databases for studies reporting quantitative findings on use of services by people living alone with cognitive impairment; 33 studies met inclusion criteria. Nine countries were represented, all high-income economies. Race/ethnicity data was reported in just five studies, and only one included a majority of racial/ethnic minorities. Overall, people living alone with cognitive impairment appear to use health services at similar or lower rates compared to those living with others; however, LTSS use is higher among people living alone. Representation of non-white participants was poor, but the evidence available suggests that among racial/ethnic minorities with cognitive impairment, there is no difference in LTSS use between those living alone and living with others. Findings highlight inconsistencies in access to and use of essential services by older adults living alone with cognitive impairment. As the populations of the US and other high-income countries become both older and more diverse, with increasing numbers living alone, researchers and service providers must consider the specific needs and preferences of this population.

